# A mixed‐method study of pain management practice in a UK children's hospital: identification of barriers and developing strategies to maintain effective in‐patient paediatric pain management

**DOI:** 10.1002/nop2.33

**Published:** 2015-10-01

**Authors:** Kate Beckett, Ellen M. Henderson, Sarah Parry, Peter Stoddart, Margaret Fletcher

**Affiliations:** ^1^ Faculty of Health & Life SciencesUniversity of the West of England, BristolBristolUK; ^2^ Louis Dundas Centre for Children's Palliative CareInstitute of Child HealthUniversity College LondonLondonUK; ^3^ Acute Pain ServiceUnited Hospitals Bristol NHS Foundation TrustBristolUK; ^4^ Acute Pain ServiceUnited Hospitals Bristol NHS Foundation Trust & University of BristolBristolUK; ^5^ Faculty of Health & Life SciencesUniversity of the West of England, Bristol & University Hospitals Bristol NHS Foundation TrustBristolUK

**Keywords:** pain, pain management, inpatient, post surgical, efficacy, nursing, pain service, hospital

## Abstract

**Aim:**

To assess Acute Pain Service and paediatric pain management efficacy in a UK specialist paediatric hospital to inform wider recommendations for future sustainability.

**Background:**

UK paediatric acute pain services vary. Although comprehensive pain management guidelines exist, consensus on the best model of care is lacking. Worldwide, medical and pharmacological advances and rapid patient turnover have increased the challenges of managing hospitalized children's pain. Simultaneously nurses, who deliver the bulk of pain management, have experienced reduction in skill mix and training opportunities. Specialist Acute Pain Services have evolved to meet these demands; their overall efficacy is unknown.

**Design:**

This mixed‐methods study explores pain management practice at a UK paediatric hospital to assess current efficacy and future sustainability.

**Method:**

A 2013 case note review of all Acute Pain Services referrals over 14 days were compared with an interval sample of concurrent non‐referred inpatient children; seven semi‐structured interviews were conducted with a range of clinical staff.

**Results:**

Twenty‐two referrals of 15 children were made; 15 comparison children were identified. All 30 children (100%) were appropriately referred/non‐referred. Acute Pain Services cases experienced higher pain levels, were more likely to have long term conditions, longer hospital stay and repeat admissions. Three key themes emerged through interview analysis: ‘addressing pain’, ‘changing contexts’ ‘pain as an “expert” skill’. Increased specialization, reduced clarity between different pain modalities and decreased training opportunities had resulted in potentially unsustainable APS dependence.

## Introduction

Despite paediatric acute pain management guidelines (Royal College of Nursing 2009, Habich *et al*. 2012, Howard *et al*. 2012, James 2014, Stevens *et al*. 2014) current provision remains inconsistent and sometimes inadequate (Royal College of Nursing 2009, Habich *et al*. 2012, Howard *et al*. 2012, Stevens *et al*. 2014). Paediatric pain has historically been undertreated (Royal College of Nursing 2009, Habich *et al*. 2012, Howard *et al*. 2012, Duncan *et al*. 2014, Stevens *et al*. 2014) and many hospitalized children worldwide still experience unresolved pain (Royal College of Nursing 2009, Howard *et al*. 2012, Duncan *et al*. 2014, Stevens *et al*. 2014). In the UK, the need for specialist Acute Pain Services (APS) was recognized in the 1980–1990s leading to local service developments (James 2014). In the USA, pain services have evolved to meet the demand in most major hospitals (Verghese & Hannallah 2010). However, these teams now face unprecedented challenges due to increasing demand, reconfiguration of care, changes to health care funding, improved survivability and heightened patient acuity (Department of Health 2004a, Verghese & Hannallah 2010). Different strategies have evolved in response to these changing needs; yet there is little consensus on the best model for managing paediatric acute pain and even well established and resourced services struggle to establish optimal care (Royal College of Nursing 2009, James 2014). This study of the APS and pain management at one UK specialist paediatric hospital provides a local window into a subject of international significance and widens debate on issues and possible solutions to improve management of hospitalized children's pain.

Pain is a common feature of paediatric conditions requiring hospital admission, but individual factors effect children's pain experience and response (Royal College of Nursing 2009). Children frequently cite pain as the most distressing aspect of disease or hospitalization (McCleary *et al*. 2004). While well managed pain can lead to improved outcomes, faster recovery and shorter hospital stay (Curtis *et al*. 2012, Howard *et al*. 2012)and untreated pain can lead to long‐term physiological and psychological effects (Royal College of Nursing 2009, Curtis *et al*. 2012, James 2014). Effective multi‐disciplinary individualized pain management is therefore essential (McCleary *et al*. 2004, Royal College of Nursing 2009, Duncan *et al*. 2014, James 2014). Pain trajectories and treatment modalities vary and require different resources and skills. Acute pain is defined as ‘pain of recent onset and probably limited duration, usually having an identified temporal and causal relationship with injury or disease’ (James 2014). In the UK, children's acute pain is generally managed by different teams than pain arising from chronic or persistent conditions and palliative care (Shum *et al*. 2012).

Caring for children in pain can be challenging and requires experience and training (McCleary *et al*. 2004, Ellis *et al*. 2007, Howard *et al*. 2012). The bulk of pain management is delivered by nurses (Royal College of Nursing 2009) who, in the UK as in many other countries, are experiencing significant workforce cuts particularly among senior staff (Ellis *et al*. 2007). Factors including; a complex array of assessment tools (Wong *et al*. 2012), advances in pain pharmacology and technology, use of off licence medicines (Department of Health 2004b) and numbers of children with complex conditions have increased the need for specialized services to maintain clinical safety and governance (Royal College of Nursing 2009, James 2014). Furthermore, anecdotal evidence suggests simple or behavioural pain management techniques are becoming underused. In the UK, the National Service Framework for the treatment of ill children (Department of Health, 2004a) recommends regular audit of children's pain management. However, systematic evaluation of APS effectiveness is difficult due to variations in models of care (James 2014), professional boundary, organizational culture and small data sets (Habich *et al*. 2012, Duncan *et al*. 2014). There is consequently a risk that current services may not be effective in managing changing or future needs.

The lead clinicians for the APS at a UK specialist paediatric hospital (‘the hospital’) were concerned their service was not best used and initiated this collaborative study to explore: (1) development and current use of the APS; (2) barriers and facilitators to pain management; and (3) apparent effectiveness of pain management to inform recommendations for improvement and future sustainability.

## Method

The hospital serves a large mixed urban/rural region and takes national referrals for specialist care. Our mixed‐methods study of current pain management was completed following formal approval by the hospital audit committee, having considered ethical and practical implications. Ward managers were informed of the study aims and processes and asked to disseminate this information to all staff. Posters containing study information were placed at prominent locations in all clinical areas. The study was supported by the specialist APS leads (consultant paediatric anaesthetist and specialist paediatric pain nurse) and hospital managers. It was funded by the University of the West of England, Bristol.

### Quantitative data

A prospective snapshot of referrals (cases) to the APS, from all clinical areas, was completed over 14 consecutive days in September 2013. The aim of this review was to establish what the current rate of use of the APS was and how effective specialist intervention was in managing children's pain. Each ward was provided with an activity log, securely stored and accessible to staff only, to record referrals during this period. A referral was defined as ‘any approach to the APS either by phone or in person with the aim of securing advice or clinical support’. Study researchers visited all areas daily to ensure referrals were correctly logged. Reflective diaries of daily encounters with ward staff were kept by the study team.

The activity log was cross‐referenced with a separate APS referral log. We allocated unique, anonymizing, identifying codes (UIC) to each referral indicating location, child's initials and referral number. Identifiable data (for tracking purposes) were stored in a separate locked cabinet. Patient consent was not required for this element of the study. Following the 14 day referral period, children's medical notes were reviewed and case information extracted as in Table 1.

**Table 1:** Data extracted from review of medical and nursing notes

Brief medical history Current episode diagnosis and treatment Age and gender Communication issues Length of stay Frequency and pattern of analgesic prescription and administration Types of pain management used (e.g. Patient Controlled Administration pump (PCA), oral, intravenous, behavioural) A full history of reported pain and pain scores on and during admission Pain assessment tool used and frequency of assessment Nursing or medical record of pain management interventions or issues Outcome of intervention (pharmaceutical or other) on pain scores

Cases provided information about the appropriateness of known APS referrals and subsequent pain management, but not about pain management by non‐specialist practitioners or appropriateness of non‐referral, i.e. was pain better managed by referral? Comparison children enabled us to address these questions through a case note review of an equal number of children (hospitalized at the same time as Cases) with conditions where pain was a probable symptom but who were not referred.

Comparison patients were identified through interval sampling from a list (generated by hospital data analysts) of all hospital in‐patients over the same 14 day period. Children with conditions where pain was unlikely to be a significant symptom or with unknown diagnosis were omitted. The remaining children were randomly ordered and cases selected at the appropriate interval to generate an equal number to Cases. A UIC was allocated to each comparison child and the equivalent data extracted. Appropriateness of non‐referral was based on clinical data, pain score trajectory, pharmacological and other pain relieving methods and fit to the existing APS referral algorithm. All cases were reviewed by two experienced clinical researchers until consensus on appropriateness of referral/non‐referral was reached.

### Qualitative data

To add breadth and aid interpretation of these data seven semi‐structured interviews with clinical staff were conducted by EH & KB. The objective of these interviews was to establish the barriers and facilitators to effect management of children's pain both locally and within discipline to establish what, if any changes needed to be made to the service to improve pain management provision. The interview sampling frame was devised to ensure perspectives of a range of nursing and medical staff from different settings and levels of seniority. We purposively sampled nurses at junior and senior levels through convenience sampling. A similar approach to recruitment of medical staff proved ineffective so a key senior clinician circulated a direct invitation (containing study information and researcher contact details) in his team. In addition, the two specialist APS practitioners were approached directly. The interview topic guide was based on our literature review and study aims and objectives. It included questions relating to APS access or use, barriers and facilitators to pain management and suggestions for service improvement. Interviews were undertaken at the participants’ place of work in a quiet and private location with no other people present. They lasted between 10‐30 minutes. Interviews were audio recorded, anonymity was assured and signed consent obtained for recording and subsequent use of the data.

All interviews were transcribed verbatim and checked for accuracy by EH and KB. Contextual notes and observations were recorded following the interview, no repeat interviews were deemed necessary. Interviews were coded using NVivo 10 software and thematically analysed according to the methodology outlined in Braun and Clarke (2006) for the development, identification and description of themes.

KB and EH completed cycles of listening, transcription and reading to immerse themselves in the data. This process informed development of a coding frame to assist with organizing and analysing the data. Further cycles of coding assisted in refining the codes and identifying emergent patterns, relationships and themes. These themes were subject to continuous discussion and clarification among the research team. The resultant findings were scrutinized by the two specialist APS practitioners interviewed in the study to ensure their views and perspectives were reliably represented and to maintain rigour of the analysis. In reporting the findings of this qualitative component quotes are attributed to profession and seniority only, pain specialist and other practitioner views are also combined to maintain anonymity (in view of the small numbers involved).

## Results

### A. Notes review

During the 14 days 22 referrals were made pertaining to 15 individual children (Cases). Fifteen Comparison children were consequently selected. Staff universally recognized this as an exceptionally quiet period throughout the hospital and that referrals form a small part of the APS workload. Case and Comparison children's characteristics are summarized in Table 2.

**Table 2:** Case and comparison children characteristics

Characteristic	Case (*N *=* *15)	Comparison (*N *=* *15)
Age years: Mean (range)	9 (<1–16)	8.7 (<1–16)
Gender	M:F 11: 4	M: F 9: 6
Analgesic route (PCA/NCA = patient/nurse controlled analgesia)	PCA/NCA: 14^a^ Oral: 1	PCA/NCA: 1^b^ Oral: 14
Maximum pain score: Mean (0–10 scale)	6^c^	2.8 (87% ≤ 5)
Duration of stay in days: Mode (range)	6 (range 1–194)	1 (1–5)
(Non) Referral appropriateness	15 (100%)	15 (100%)

a After APS review: PCA = 15.

b Out of hours PCA management by non‐APS anaesthetist.

c Excluding 4 with pain score ‘0’ pre and post APS review.

Children's age and gender were comparable between groups but 12 Cases had long term complex conditions and had already seen the APS during this admission (five Comparison children had chronic conditions but none had required APS intervention during this admission). Case children's average pain scores (omitting those with pain scores of ‘0’ pre and post review) were higher and the length of stay was also longer. Their notes suggested that recent prolonged or repeat hospitalization and many had persistent pain issues. Three cases had communication issues (two due to learning disability: one due to exceptionally low mood) as did two Comparison children (one learning disability: one pre‐verbal).

All Cases referred to the team were considered appropriate, they followed local guidelines for management of children with PCA (14 children) or had appropriate analgesic escalation before referral (one child). However, nearly a third of ‘referrals’ were for review of PCA (without evidence of raised pain scores pre or post review). One child was urgently referred to multiple practitioners simultaneously due to worsening condition, irritability and distress. This extraordinary referral for diagnostic purposes adhered to PCA management guidelines, to exclude pain as a principle problem. Case children generally received multimodal analgesia, including oral and PCA routes, but simpler analgesic use was generally below prescribed maximum levels. Pain scores fell in nine Case children following APS review. For two cases this involved addition of Diazepam for pain caused by muscle spasm (four Cases had no pain before or after review and one referral was for other reasons – see above).

Comparison children tended to be in hospital for relatively short periods for acute conditions, chronic condition flare or surgery. Their pain was generally successfully managed by ward staff using a range of simple or stronger oral analgesics. In two children, there was a delay between recording a raised pain score and analgesic administration. One child was receiving PCA which was managed appropriately by an anaesthetist responsible for out‐of‐hour's provision. Reporting of these children's pain scores was generally good. We had no evidence to indicate that interaction with the pain service would significantly alter the pain management in these cases. Behavioural pain management techniques were rarely recorded. We found little evidence pertaining to early discharge advice or prescription postsurgery.

### B. Qualitative data findings

Seven interviews were completed with a range of clinical ward and APS staff (Table 3 lists participant characteristics). Three major themes emerged through analysis of the interview data: ‘addressing pain’, ‘changing contexts’ and ‘pain as an expert skill’.

**Table 3:** Interview participant characteristics (*N *=* *7)

Position	Medical consultant (1), Medical registrar (2), Senior Nurse (2), Junior nurse (2)
Duration of service	0–2 years (1), 3–10 years (3), 11 + years (3)
Gender	Female (6): Male (1)
Age	Not formally recorded but selecting junior and senior staff effectively achieved a wide spread

#### Theme 1: Addressing pain

Our participants all desired to eliminate children's pain which was a major factor in most of their patients’ care. Unresolved poorly managed pain was distressing for the child, their family and clinical staff:

we've had so many days and nights where she's [a patient] just been crying in pain … it's not hard nursing wise it's just you feel useless because you can't take away the pain and that is what you feel, that is my job I want to be able to (Junior nurse)

Managing children's pain could be difficult and required knowledge, experience and skill. Being able to focus on pain and ignore other demands was an advantage; some staff felt they lacked the necessary time and the APS was better able to do this properly:

They [the APS] can offer new things that we won't think of or, you know, they can spend the time talking to the families about the pain. Because we have to cover nutrition, blood count, pain, outcome, when do I go home, everything! So the patients, they know that when they [the APS] come in it's the moment to talk in detail about pain, about all of it. (Doctor)

Pain resulting from some conditions and in some children was harder to manage, for example the pre‐verbal or learning disabled child:

I find it really difficult to care for the children with epidurals with special needs who can't communicate … it is really difficult to assess their motor block of their epidural … it's always hard to assess whether they are in pain. (Junior nurse)

I suppose I mean its difficult adolescents can tell you where the pain is they can tell you how much pain they are in, you can score them … with a younger child it's a lot harder to assess are they crying cos they are anxious? Are they crying cos they are in pain? Are they crying, you know, just because they don't like you? (Junior Nurse)

Family coping or parenting styles could also impact on the child's response to and experience of pain (and on provision of care):

When …they are in pain and they can't express that pain the parents get quite frustrated and quite angry and the child then becomes cross and you are trying to unpick all of that and give the right support. So that can be a difficult situation to manage. (Senior nurse)

The APS evolved at a time when managing pain was simpler but was poorly understood and under prioritized. They had raised the profile of pain as ‘the fifth vital sign’ and offered an accessible source of clinical support, training and reassurance that practitioners had ‘got it right’. However, their remit had expanded rapidly in line with medical, pharmacological and technological developments. Originally a specialist team to manage children's postoperative and procedural pain, the APS was now perceived as leading on all aspects of in‐patient pain (with responsibility for clinical standards, teaching and support). However, the team size had not altered:

It's difficult with there only being one anaesthetist on call and one clinical nurse specialist (Junior nurse)

Access and use of the APS varied between wards. Some areas such as the Accident and Emergency department and intensive care felt they had other intensivists ‘on hand’ who they used for pain management support.

#### Theme 2: Changing contexts

The complexity of conditions and degree of pain experienced by children was felt to have had increased over the years and distinctions between acute, chronic and palliative pain (and responsibility for their management) appeared to have become blurred. The APS had responded by expanding their remit and developing guidelines and competencies to assist and regulate practice. While the majority of children had manageable finite pain (as a consequence of acute conditions or surgical procedures) increasingly a significant proportion presented with conditions not previously considered treatable or survivable:

A lot of what we see now, I'm not seeing all the basic bread and butter. What I'm seeing is the complex stuff more and more and I think that is where stuff has changed. Children with certain chronic diseases are living longer and there are some that have had surgery at a younger age and we're now seeing the outcome of that and secondary level surgery. (Senior Nurse)

The APS was increasingly regarded as responsible for managing not only perioperative or acute pain but pain resulting from medical and oncological conditions as well:

We tend to use the pain team for a variety of patients in the haematology oncology scenario so BMT patients with mucositis and then needing patient controlled or nurse controlled analgesia. And also, kind of some of the sickle cell crisis admissions probably as well when I'm on call. So those are the types of patients that may have ended up on regular intermittent IV analgesia which isn't working and then referred to the pain team for continuous IV infusions. (Doctor)

The APS was essential in managing these children's pain but they were unavailable out of hours:

When they [the APS] are around you know, Monday to Friday nine to five, brilliant…really accessible. But it's … the unsociable hours when it's not (Junior Nurse)

Responsibility for out‐of‐hours management of complex pain appeared unclear:

The patients have become almost too complicated. For some of the registrars to feel happy managing overnight, or at weekends and … it's come back to us and because we don't have a resident palliative paediatric pain team we're expected to then take it back over. (Doctor)

Turnover of children with simpler conditions or treatments had become more rapid increasing the need for parents to manage pain at home. Those in hospital for longer were generally undergoing complex procedures, were frequent attenders or had life limiting conditions. Increasing numbers had contact with the pain service during their admissions. Many had more persistent forms of pain and complex pain management histories. They were universally acknowledged as hard to manage and their suffering was sometimes distressing. Specialist interventions, requiring creativity, experience and skill were required to manage their pain:

And then there is the oncology on the edge of the palliatives. So very complex they are going beyond any guidelines very individualized care, very distressing times for everybody. And pain management is both theoretical and clinical, but it is also an art. (Senior nurse)

We are having so many teenagers … their compliance or their engagement with the treatment is sometimes, they want to do what they think is right and they don't listen. I want this and this and this and this after and I want it after, as a bolus and this one is an infusion and this one with this one together … they have been a long time in hospital they want to control everything …it takes a lot of work to make changes or to get them to understand, no you can't have this and this together. It's not safe (Doctor)

Expanding use of pain management equipment such as the patient controlled analgesia (PCA) pump and of non‐licensed medicines had also increased the specialization of pain. Simultaneously resource constraints, increase in demand and downgraded skill mix were leading to fewer opportunities for training, clinical supervision, mentoring and support for newer staff:

Currently … staffing levels don't give you much leeway or much flexibility to allow that teaching … I think we did used to manage the children's pain probably more effectively maybe when the pressures weren't as … they are now … and you had time to spend with junior staff to teach them. (Senior nurse)

The fundamental barrier is … the pressure that the ward staff have in terms of just delivering their own care and having protected time to come to training …there is a huge pressure to get patients … through hospital very quickly and therefore you can't just stop… to go and teach (Doctor)

#### Theme 3: Pain as an ‘expert’ skill

These changing circumstances combined with drives to improve quality and safety had resulted in some practitioners becoming less confident and lacking trust in their own and other's clinical judgement. Relinquishing responsibility to ‘the experts’ was sometimes considered a safer option:

We are not that comfortable with some drugs and we don't know how to use them or we don't know risks. And … the rest of the staff is not quite sure if you prescribe them cos you know are you sure? … they [the APS] are very expert and I am …. not maybe the best person to do it. And everybody feels more comfortable to get the experts to do it. (Doctor)

But led to unsustainable demand on the APS who struggled to manage their extended remit:

It's almost like … a snowball as time has come down the hill. And whether that is kind of quite nice to reflect that they use [the APS] or whether or not there are other issues that have poured into it. Such as you know … increased patient population and acuity of patients etc. and complexity. But the service feels like it's mushroomed, almost out of control. (Senior nurse)

However, a range of factors determined patterns of APS use. Those with sufficient experience, skill and training remained confident in their ability to independently manage children's pain (especially children who conformed to their own speciality). ‘Outlying’ children whose condition or analgesic route fell outside their expertise could be harder to manage:

I'm quite used to people … with PCA's and morphine … but I know that some of the other nurses [find it] a bit more challenging … because we don't see it we more usually get like bronchs and asthma on the ward and our first line drug is … Paracetamol and Brufen … we don't tend to give morphine out or like some of the stronger drugs as a kind of pain basis here (Senior Nurse)

Participants perceived their access to the APS as varied. Some areas received APS ‘rounds’ due to large numbers of postoperative cases while others (particularly medical wards) felt they referred on a needs basis. Some junior practitioners in areas with ‘routine’ APS rounds depended on them to provide guidance and support in most aspects of pain management:

I probably use the APS or our ward uses the APS on a daily basis…for all sorts of things…they are … there on hand to give advice and speak to them about where to go next … I use them all the time. (Junior Nurse)

But ease of access to the APS could result in other causes of irritability or distress being overlooked:

In a child who can't communicate who is distressed, again we often by default end up giving them analgesia. Again I'm not sure it's always the correct thing. (Doctor)

In areas where APS access was felt to be less predictable, clinicians generally rated their practice as good but felt unable to provide effective pain management for some children. Broader experience elsewhere had enabled some practitioners to develop useful competencies and skills which reduced the fear associated with managing children's pain:

I think what I found really helpful in my old trust is we had like taster days and we had like skills specifically for pain to be signed off in our first year preceptorship and I got signed off in PCAs and epidurals so I was used to working with them and I wasn't scared (Junior Nurse)

I have a very practical view of pain because where I come from we used to do everything ourselves. So I'm not scared of giving added morphine because I've done it before. I'm used to prescribing infusions. I used to do all that part. So I'm not scared (Doctor)

Limited availability of analgesia – through inadequate prescription or withdrawal of drugs licensed for use in children (e.g. codeine) – was also cited as a barrier to effective pain management. Only one participant referred to behavioural pain management techniques (the majority of interventions described were pharmaceutical). This may imply lack of training, lack of application in this setting or simply that recording of such techniques is poor.

### Overview

All accounts suggested the APS were considered key in raising standards, guideline development, supporting staff and managing pain in children with complex conditions:

We have … very challenging patients. Like globally super complicated. I think they (APS) have had a key role on that because, you know, maybe the patient has some problems in their list. But for them pain is number one, top of the list. (Doctor)

But many expressed ambivalence towards what they perceived of as increasing specialization of pain and practitioner dependence on the APS. This was reinforced by imbalance between the APS capacity to provide educational and clinical input and by service demands:

I think the nursing staffs on the wards have been de‐skilled… we used to manage … the pain to the children … I think you've put in a specialist service and the nursing staff pull back. (Senior nurse)

There is this dependency and people are very stretched doing other things and so things tend to get compartmentalized. And pain, well, there is a pain service… (Doctor)

## Discussion

The need to manage hospitalized children's pain effectively is paramount (Ellis *et al*. 2007, Royal College of Nursing 2009, Howard *et al*. 2012, James 2014) and specialist practitioners are important to improve standards of pain management (McCleary *et al*. 2004, Ellis *et al*. 2007, James 2014). Pain is clearly a major issue for many UK hospitalized children and even children referred to the APS may experience high levels of pain (in this case, the mean maximum pain score was 6/10, excluding those with no pain before or after review). This study suggests that current provision in this UK hospital is generally effective. Our study evaluated the current use of APS in one children's hospital and found that the APS is a key in maintaining standards and managing more complex pain. This limited sample suggests referrals to the APS are appropriate and children not referred do not appear to experience more pain as a result (although delay in responding to raised pain scores needs to be addressed). The current use of the service seems to be appropriate and effective in the management of pain in the children that are referred to them.

However, we also demonstrate the changing context of NHS paediatric care and how services designed for one purpose frequently evolve to fill many more. This APS clearly provides (both in reality and in the minds of other practitioners) a generic pain management role in the hospital. How this relates to or overlaps with services responsible for paediatric palliative and chronic pain remains unclear. Clarification of these services remit and more co‐ordinated service development may be of benefit.

However, in this study paediatric pain management effectiveness is largely sustained by specialist and senior staff while development of junior staff competence is limited and inconsistent. This is not unusual, successive studies demonstrate how escalating NHS patient and organizational demands have impacted on capacity for training and mentoring (Ellis *et al*. 2007). Furthermore, less than 1% of university clinical training focuses on pain identification and management despite its ubiquity as a symptom of disease or treatment. (Howard *et al*. 2012). The risk of this is that frontline or junior staff providing 24 hour care may increasingly lack the skills, knowledge or confidence to provide immediate relief from pain, address different pain modalities e.g. diazepam for spasm and use ‘simple’ analgesics optimally. The use of behavioural pain management was not explicitly explored in this study. However, the extremely limited reference to such alternatives supports their likely underuse, despite evidence of their effectiveness (Curtis *et al*. 2012). It is possible that the increasing specialization of pain reported also influences the use and development of other simpler interventions or narratives. Given links between psychosocial factors and children's response to and experience of pain, it is important that these alternative approaches are not neglected (Williams *et al*. 2012) and that pain management remains holistic and multi‐disciplinary (Royal College of Nursing 2009, Howard *et al*. 2012, Williams *et al*. 2012, Stevens *et al*. 2014).

This study supports the concerns of others that specialist teams may contribute to de‐skilling generalist practitioners if their simultaneous development and training is neglected (Castledine 2004). This is particularly the case for more junior staff. Practitioner's primary objective is to effectively and safely manage children's pain and they will naturally adopt strategies which support this aim. While this hospital appears to have effective means of managing children's pain (as demonstrated by the quantitative data) the qualitative data suggests considerable potential for this model to become unsustainable. It is unlikely that these issues are restricted to this one service (Royal College of Nursing 2009, Duncan *et al*. 2014, James 2014).

While barriers to enhancing pain management practice are well documented (Ellis *et al*. 2007, Royal College of Nursing 2009, Habich *et al*. 2012, Howard *et al*. 2012) studies elsewhere suggest strategies’ which may be transferrable to the UK setting. For example, McCleary *et al*. (2004) in Canada found that identifying and training paediatric pain resource nurses (PRNs) for each clinical area was a ‘key element in a comprehensive programme to improve pain management’. Link Nurses occupy a similar position in the UK. However, while these roles can improve some aspects of pain management knowledge and practice their effectiveness is reduced without simultaneous organizational support for dedicated time and development of leadership and change skills. Another Canadian team effected significant improvements in pain management through trial of a multidimensional knowledge translation intervention called ‘Evidence‐based Practice for Improving Quality (EPIQ)’ which integrated evidence, local contextual knowledge and facilitation (Stevens *et al*. 2014). Guidelines are another important means to improve the effectiveness and consistency of care. However, adherence depends on frontline staff involvement in their development and ongoing training and monitoring of their use (Habich *et al*. 2012). In Australia, Boyd & Stuart (2005) found that using a structured pain assessment tool and nurse initiated oral analgesia protocol could significantly reduce time to administration and increase analgesic cover in children presenting with mild to moderate pain. Such initiatives may also translate well but not in isolation, multifaceted strategies and sustained ongoing organizational, interdisciplinary and ‘grassroots’ support are essential to practice improvement (Ellis *et al*. 2007, Habich *et al*. 2012, Duncan *et al*. 2014, Stevens *et al*. 2014). Further means to support pain management include: (1) prescription of a range of analgesia to meet all potential needs and permit timely adjustments according to clinically assessed need and (2) considering regular multimodal ‘round‐the clock’ analgesia or increased background dosage rather than prescribing on an ‘as‐required basis’ (McCleary *et al*. 2004).

Our study supports the notion that children's pain management has become increasingly complex and calls for more systematic research of local practice to inform specification of the best model of care. Local audits such as this can contribute much needed information about how services are organized and the strengths and limitations of current practice. While we encountered some initial problems this study did not confirm reported difficulties in research in this area (Habich *et al*. 2012, Duncan *et al*. 2014, James 2014).We found that practitioners desired to improve services but lacked capacity to initiate or engage in the process. Collaborations between academic and clinical settings can be of mutual benefit in combining theory and practice to elicit practical solutions with potential for implementation in real world settings (Flottorp *et al*. 2013).

Extrapolation from the study findings and the literature suggest a series of wide ranging suggestions for policy, practice and research (Table 4).

**Table 4:** Implications for policy, practice and research

Policy
Consensus on the best model of paediatric pain management should be agreed. Specialist paediatric pain services require capacity to provide 24/7 cover (DOH 2004a) and to deliver both clinical and educational aspects of their role APS capacity and resources should be subject to regular review. Pain assessment and management should be accorded more priority in university clinical training Training and ongoing support of junior staff and senior staff capacity for mentoring and clinical supervision should be prioritised and protected. Further reductions in skill mix and frontline staff may impact on effective paediatric pain management; these implications should be carefully considered Reinforcing organisation wide pain management competence and skills is essential to ensure the effectiveness, safety and experience of care. Integrating paediatric chronic, acute and palliative pain services may be necessary to permit knowledge and resource sharing and meet the changing context of care.
Practice
The role and scope of the specialist APS needs to be clearly identified Pain management should be supported by clear and simple APS referral criteria Children universally recognised as likely to have complex pain needs should be identified early and their care supervised by a specialist pain team Existing practitioner capacity for management of moderate to severe pain should be supported and developed through a programme of ongoing training and dissemination including; Post registration training and assessment of key pain management competencies Identification and development of Pain Resource Nurses (or Link nurses) in each area Monthly pain specialist practitioner/PRNs meetings Expanded opportunities for multi‐disciplinary education and training Case studies (regarding hard to manage groups or recent cases to improve knowledge and understanding) Further teaching and support in behavioural pain management techniques The effectiveness of training or guidelines can be improved through use of multi‐dimensional strategies such as EPIQ (Stevens *et al*. 2014) and frontline staff involvement in guideline development Prescribers should be equipped with knowledge to prescribe a range of analgesia and if drugs are withdrawn (e.g. codeine) other options should be fully explored (Wong et al 2012) Practitioners should be encouraged to explore other causes of distress There is a need for better communication between disciplines and shared acknowledgement of each other's expertise and difficulties in managing children's pain Formal systems for reviewing children in ‘outlying’ wards are essential
Research
Prospective longitudinal studies to evaluate alternative pain management models to improve understanding of variations in care and best models of practice. Factors affecting the efficacy and timeliness of simple analgesic administration Current usage and availability of behavioural pain management techniques Parental preparation for managing children's post‐surgical pain at home National variations in responsibility for PCA/NPA management should be explored Practitioner usage of the correct assessment tool for children's age and developmental stages Pain management strategies and effectiveness in intensive areas such as the ED and A+E Patient and carer perspectives on local pain management practice

### Study strengths and limitations

We found little evidence from medical records or interviews on how parents were supported to manage their children's postoperative pain at home. This is important given increasingly rapid turnover and evidence that children experience significantly more pain at home (Rony *et al*. 2010, Shum *et al*. 2012) and that many parents lack the ability to assess and make decisions about appropriate analgesia especially postoperatively (Knutsson *et al*. 2006). Limited recording of practitioner choice of pain assessment tool (against child age and developmental stage) also prevented evaluation of their appropriateness. Pain scores recorded by staff are known to differ from parental or child estimations of pain (Knutsson *et al*. 2006), while the perspectives of staff delivering care are important this study would have benefited from children's and parents views too.

The number of interviews and cases in this audit were small due to funding, time constraints and organizational pressures. Convenience sampling and self–selection of interview participants may have resulted in perspectives which differ from other practitioners. This study should be replicated in other institutions to assess how the results would compare in different settings with differing local and other considerations. The major themes in the qualitative data consistently emerged through thematic analysis and were further supported by researcher reflective diaries of encounters with other staff. Deeper analysis was limited by participant numbers and interview brevity. However, use of qualitative and quantitative measures improved the reliability and breadth of our findings. Analyses of the data were performed by EH and KB to reduce potential APS bias in reporting the results. However, the transferability and reliability of the findings was tested by asking two interview participants to comment on preliminary analysis. This study did not permit evaluation of Emergency department (ED) and Paediatric intensive care unit (PICU) pain management (no children were referred during the snapshot and no staff participated in interviews). Pain management in ED and PICU is extremely important due to levels of pain experienced in these areas and evidence suggesting sedation may sometimes obscure pain (Hall 2012).

## Conclusion

Providing effective children's pain management is essential but challenging due to increasing complexity of conditions, demands and pressure on services. Many strategies have evolved to optimize paediatric pain management and have contributed to improvements in standards of care. Evaluation of one such local strategy and research into other models of care can inform future development of children's pain services. The advantages of specialist APS in raising standards and improving patient care are clear. However, without forward planning and simultaneous investment in training there is a risk that pain becomes increasingly specialized, that responsibility for managing other forms of difficult‐to‐treat pain becomes blurred and that front‐line staff lack the ability to provide timely effective care. These issues are unlikely to be restricted to the UK context. Future provision of effective safe pain management will depend on valuing and developing all practitioners’ knowledge and skills rather than allowing some to become disempowered and deskilled (Castledine 2004, Duncan *et al*. 2014). Future integration of paediatric chronic, acute and palliative pain services may be necessary to ensure knowledge and resources can meet the changing landscape of paediatric care.

## Afterword

Many of the recommendations in Table 4 have been locally discussed and/or implemented since this study.

## Conflict of interest

EH, KB, MF completed the submitted work as employees of the University of the West of England; SP, PS are UHBristol employees and KB, EH, MF held honorary contracts. No other relationships or activities could have influenced the submitted work or caused any conflict of interest.

## Author contributions

KB, EH, SP, PS, MF made substantial contributions to the conception and design of the work; KB, EH, MF made substantial contributions to the acquisition and analysis of the data; KB, EH, SP, PS, MF contributed substantially to the interpretation. All authors contributed to drafting the work, revising it critically and approved the final version to be published.

All authors have agreed on the final version and meet at least one of the following criteria [recommended by the ICMJE (http://www.icmje.org/recommendations/)]:

- substantial contributions to conception and design, acquisition of data, or analysis and interpretation of data;
- drafting the article or revising it critically for important intellectual content.
